# A new species of *Asecodes* Förster (Hymenoptera, Eulophidae) and first record of *A. reticulatum* (Kamijo) from China, with a key to Chinese species

**DOI:** 10.3897/zookeys.1049.65964

**Published:** 2021-07-15

**Authors:** Ming-Rui Li, Cheng-De Li

**Affiliations:** 1 School of Forestry, Northeast Forestry University, Harbin, 150040, China Northeast Forestry University Harbin China

**Keywords:** Chalcidoidea, Entedoninae, natural enemy, parasitoid wasp, taxonomy

## Abstract

A new species of *Asecodes* Förster, *A. medogense***sp. nov.** is described from Tibet, China and *A. reticulatum* (Kamijo) is reported from China for the first time. A key to all known species of genus *Asecodes* in China is provided.

## Introduction

The genus *Asecodes* (Hymenoptera, Eulophidae, Entedoninae) was established by Förster (1856), but he did not include any species in it. Förster (1861) described the first two species in *Asecodes*: *A. fuscipes* Förster and *A. nitens* Förster. Ashmead (1904) designated both species as type species of *Asecodes*, and Bouček (1988) subsequently selected *A. fuscipes* as its type species. Graham (1993) synonymized these two species under *A. congruens* (Nees, 1834). Bouček and Askew (1968) synonymized *Ganahlia* Dalla Torre with *Asecodes*, Hansson (1996) synonymized *Teleopterus* Silvestri, *Metasecodes* Erdős and *Desmatocharis* Graham with *Asecodes*. Up to now, this genus contains 26 valid species worldwide: 22 species were recorded in the Universal Chalcidoidea Database (Noyes 2019), and four species were described recently by Jamali et al. (2021).

The genus *Asecodes* can be easily separated from other genera in Entedoninae by: subtorular grooves present (Figs 2, 10); having a strong and complete occipital median sulcus which reaches from the occipital margin to the foramen magnum (Fig. 21), instead of a weak fold (Fig. 22). More characters of *Asecodes* can be seen in Hansson (1994) and Hansson (1996).

Before this study, there are only three species of *Asecodes* known from China, *Asecodes sinense* (Ling) was first described from China by Ling (2000), *A. turcicum* (Nees) and *A. delucchii* (Bouček) were reported from China by Ling (2000) and Zhang et al. (2007) respectively. This paper includes five species of *Asecodes* distributed in China, *A. medogense* sp. nov. is described as new to science, and *A. reticulatum* (Kamijo) is first reported from China. A key to all known Chinese species based on females is provided.

## Materials and methods

Specimens were collected by Malaise traps and sweeping nets and were mounted on a card, or dissected and mounted in Canada Balsam on slides following methods described by Noyes (1982). Photos were taken with an Aosvi AO-HK830-5870T digital microscope or a digital CCD camera attached to an Olympus BX51 compound microscope. The quality of these photos was improved by using Helicon Focus 7 and Adobe Photoshop 2020. Measurements were made using the built-in software of Aosvi AO-HK830-5870T.

Terminology follows the Hymenoptera Anatomy Consortium (2021), and the following abbreviations are used: F1–5–flagellomeres 1–5; HE–height of eye; MS–malar space; MV–marginal vein; OOL–minimum distance between a posterior ocellus and corresponding eye margin; PMV– postmarginal vein; POL–minimum distance between posterior ocelli; SMV–submarginal vein; STV–stigmal vein; WM–width of mouth opening.

Type material is deposited in the insect collections at Northeast Forestry University (**NEFU**), Harbin, China.

## Taxonomy

### Key to Chinese species of the genus *Asecodes* (females)

**Table:** 

1	Fore wing hyaline, without infuscate transverse band, and with three stigmal hairlines (Fig. 20)	***A. delucchii* (Bouček, 1971)**
–	Fore wing with an infuscate transverse band below MV, and with two stigmal hairlines (e.g., Figs 5, 13)	**2**
2	Pedicel much shorter than F1; anterior 2/3 of mesoscutellum reticulate and posterior 1/3 smooth	**3**
–	Pedicel as long as F1 (Figs 3, 11); mesoscutellum entirely reticulate (Figs 4, 12)	**4**
3	Gaster ovate; metasoma subequal to mesosoma, shorter than head plus mesosoma (ratio length of: metasoma : head : mesosoma about 3.2:1:3)	***A. turcicum* (Nees, 1834)**
–	Gaster oblong ovate; metasoma distinctly longer than mesosoma, also slightly longer than head plus mesosoma (ratio length of: metasoma : head : mesosoma about 5.2:1:3.5)	***A. sinense* (Ling, 2000)**
4	Scape with apex of ventral margin curved smoothly in a wide arc (Fig. 11); meshes of reticulation on mesoscutum and mesoscutellum relatively coarser and larger (Fig. 12); disc of fore wing with sparse setation (Fig. 13)	***A. reticulatum* (Kamijo, 1986)**
–	Scape with apex of ventral margin curved nearly in a right-angle (Fig. 3); meshes of reticulation on mesoscutum and mesoscutellum relatively denser and smaller (Fig. 4); disc of fore wing with denser setation (Fig. 5)	***A. medogense* sp. nov.**

#### Asecodes medogense

Taxon classificationAnimaliaHymenopteraEulophidae

Li & Li sp. nov.

864260CD-27C1-5D0B-B062-2BFB5B48A0AA

http://zoobank.org/6C5E7366-1C1A-4B4E-9699-78C819C9D500

Figs 1 , 2–8

##### Type material.

***Holotype***: ♀ [NEFU; on card], CHINA, Tibet, Medog County (altitude: 1400 m), 11–18.V.2017, Zhaxi, by Malaise trap. ***Paratypes***: 1♀ [NEFU; on slide], CHINA, Tibet, Medog County (altitude: 1400 m), 15–22.VI.2017, Zhaxi, by Malaise trap; 3♀ [NEFU; 2 on cards, 1 on slide], CHINA, Tibet, Medog County (altitude: 1400 m), 6–13.VII.2017, Zhaxi, by Malaise trap.

**Figure 1.**
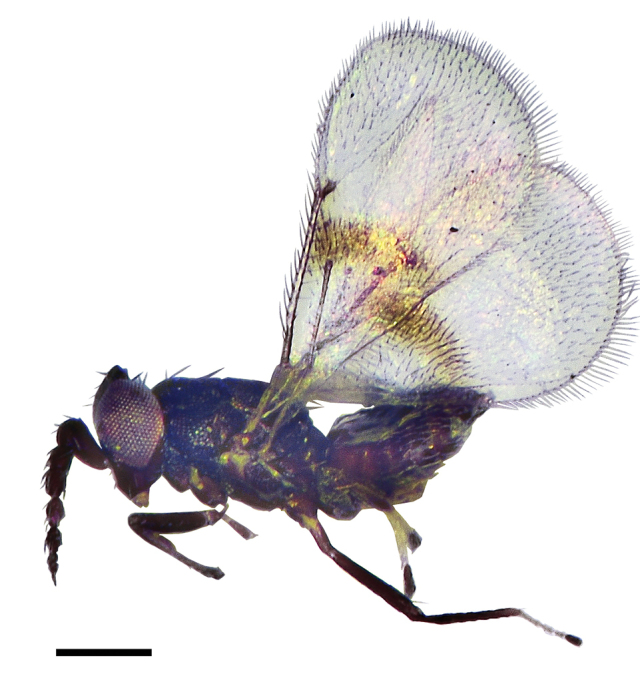
*Asecodes medogense* Li & Li, sp. nov., holotype, female **1** habitus in lateral view. Scale bar: 200 μm.

##### Diagnosis.

**Female.** Scape strongly compressed from side to side and expanded from base to apex, with apex of ventral margin curved nearly in right-angle; pedicel as long as F1; F3 distinctly paler than other segments (Fig. 3); mesoscutellum densely and entirely reticulated with small meshes; propodeum with groove along median anterior margin, without carina or plica (Fig. 4); fore wing with a complete infuscate transverse band below MV (Fig. 5).

**Figures 2–8.**
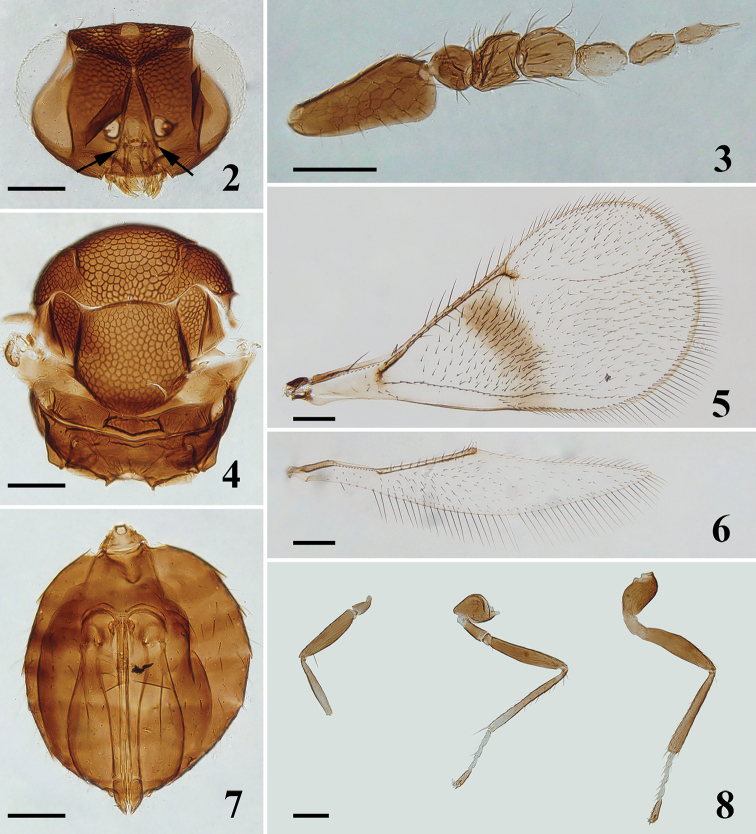
*Asecodes medogense* Li & Li, sp. nov., paratype, female, on slide **2** head, frontal view, arrows show subtorular grooves **3** antenna **4** mesosoma **5** fore wing **6** hind wing **7** metasoma **8** legs, from left to right: fore, mid and hind leg. Scale bars: 100 μm.

##### Description.

**Female.** Body length 0.8–0.9 mm. Antenna mainly dark brown, except F3 distinctly paler than other segments (Fig. 3). Vertex and frons above frontofacial sulcus metallic bluish-green, frons below sulcus golden green. Mesosoma dark brown with weak metallic blue tinges. Gaster dark brown to brown with weak metallic bronze reflections. Fore wing with a complete infuscate transverse band below MV (Fig. 5). All coxae and femora dark brown. Protibia mainly pale brown with basal part slightly daker; mesotibia mainly dark brown with apical 1/4 pale brown; metatibia dark brown. All tarsi with tarsomeres 1–3 pale yellow, tarsomere 4 dark brown.

***Head*** (Fig. 2), narrow in dorsal view. Upper face and vertex with strong reticulate sculpture, lower face with weak and irregular sculpture. Frontofacial sulcus weakly V-shaped, in an angle of about 130°. POL:OOL = 8:5. Occipital median sulcus present and complete. Inner orbits sinuate in lower part. HE:MS:WM about 3.3:1: 1.8. Malar sulcus present. Antenna (Fig. 3) inserted above level of lower margin of eyes. Subtorular grooves present. Scape reticulated, strongly compressed laterally and expanded from base to apex, about 2.1 times as long as its maximum width, with apex of ventral margin curved nearly in a right-angle. Pedicel as long as wide, and as long as F1. F1 quadrate, slightly shorter than F2 (about 0.8 times); F2 slightly longer than wide (about 1.2 times); pedicel and F1–F2 with strong and long setae. F3–F5 longer than wide and distinctly narrower than F2; F3 1.7 times as long as wide; F4 twice as long as wide; F5 narrowest, with a long terminal spine.

***Mesosoma*** (Fig. 4), 1.2 times as long as wide. Pronotum reduced, invisible in dorsal view. Mesoscutum, mesoscutellum and axillae entirely with strong reticulate sculpture, meshes on midlobe of mesoscutum and mesoscutellum small and dense (compared with *A. reticulatum*), but wider than that on lateral lobe of the mesoscutum and axillae; propodeum almost smooth; metascutellum and lateral panels of metanotum with weak and irregular sculpture. Notauli incomplete, indicated only in anterior part. Midlobe of mesoscutum with two pairs of setae. Anterior part of axillae advanced forward in front of level of anterior margin of mesoscutellum. Mesoscutellum as long as wide, with one pair of setae. Propodeum long, about 0.34 times as long as mesoscutellum, with a groove along median anterior margin, without carina or plica. Fore wing (Fig. 5) twice as long as wide. Ratio length of: SMV:MV:PMV:STV about 5.5:8.5:1:1. Speculum closed below, with two stigmal hairlines. Hind wing (Fig. 6), 5.2 times as long as wide. Legs (Fig. 8), with coxae distinctly reticulated; mesotibial spur as long as corresponding basitarsus; metatibial spur shorter than corresponding basitarsus.

***Metasoma*** (Fig. 7), gaster ovate, as long as mesosoma; petiole short, conical; first gastral tergite occupying nearly 1/4 length of gaster; ovipositor originates from about the anterior margin of second gastral tergite and slightly exserted beyond apex of gaster.

**Male.** Unknown.

##### Host.

Unknown.

##### Etymology.

The specific name is derived from the name of the collection locality of the type specimens.

##### Distribution.

China (Tibet).

##### Remarks.

*Asecodes medogense* is similar to *A. reticulatum* in having the mesoscutellum entirely reticulate; pedicel nearly as long as F1; fore wing with an infuscate transverse band below MV. The new species differs from *A. reticulatum* in having scape with apex of ventral margin curved nearly in a right-angle (curved smoothly in a wide arc in *A. reticulatum*); meshes of reticulation on mesoscutum and mesoscutellum relatively denser and smaller (relatively coarser and larger in *A. reticulatum*); disc of fore wing with more dense setation than *A. reticulatum*.

#### Asecodes reticulatum

Taxon classificationAnimaliaHymenopteraEulophidae

(Kamijo)

A56CC4C6-66D6-5CE1-B385-1FB3BE0D6F6D

Figs 9 , 10–16

Closterocerus reticulatus (Kamijo): Gumovsky 2003: 33.
Desmatocharis reticulata Kamijo, 1986: 243.
Teleopterus reticulatum (Kamijo): Hansson 1996: 162.
Teleopterus reticulatus (Kamijo): Hansson 1994: 669.

##### Material examined.

1♀ [NEFU; on slide], China, Heilongjiang Province, Yichun City, Dailing District, Liangshui Forestry Station, 28.VII.2015, Si-Zhu Liu, Xin-Yu Zhang and Xing-Yue Jin, sweeping; 2♀ [NEFU; 1 on card, 1 on slide], CHINA, Heilongjiang Province, Yichun City, Dailing District, Liangshui Forestry Station, 9.VII.2013, Guo-Hao Zu, Si-Zhu Liu and Hui Geng, sweeping.

**Figure 9.**
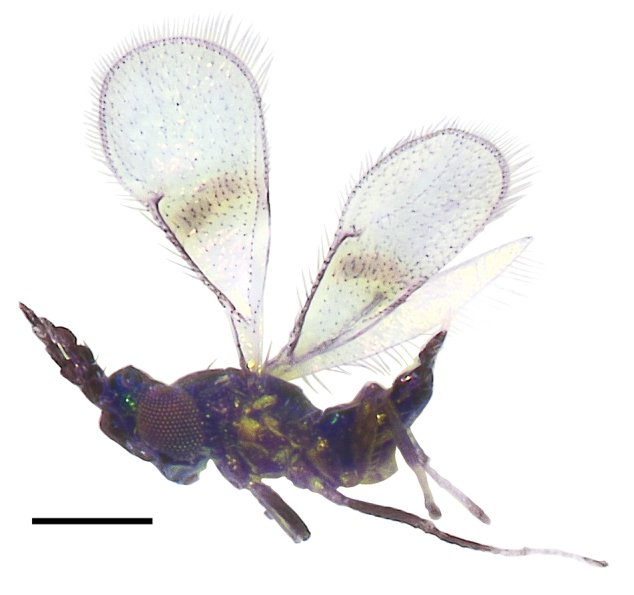
*Asecodes reticulatum* (Kamijo), female **9** habitus in lateral view. Scale bar: 200 μm.

##### Diagnosis.

**Female.** Scape compressed, with apex of ventral margin curved smoothly in a wide arc, pedicel as long as F1 (Fig. 11); mesoscutellum sparsely and entirely reticulated with wide meshes, propodeum shorter than 1/3 length of mesoscutellum (Fig. 12); fore wing twice as long as wide, and with an infuscate transverse band below MV, disc of fore wing with sparse setation (Fig. 13).

**Figures 10–16.**
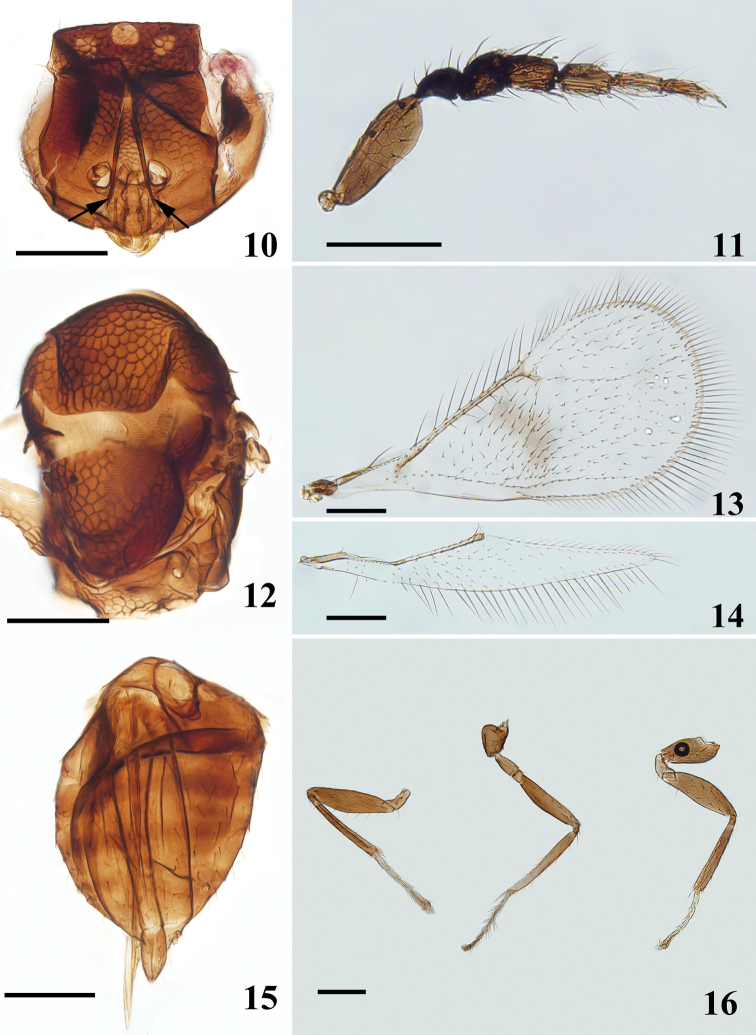
*Asecodes reticulatum* (Kamijo), female, on slide **10** head, frontal view, arrows show subtorular grooves **11** antenna **12** mesosoma **13** fore wing **14** hind wing **15** metasoma **16** legs, from left to right: fore, mid and hind leg. Scale bars: 100 μm.

##### Host.

Primary parasitoid of *Rhamphus oxyacanthae* (Marsham) (Coleoptera, Curculionidae) (Hansson 1994).

##### Distribution.

China (Heilongjiang Province) (new record), Japan (Kamijo 1986), Russia (Gumovsky 2003), Ukraine (Gumovsky 2003) and Sweden (Hansson 1994).

##### Comments.

See Kamijo (1986) for a detailed description; our specimens agree well with this description.

#### Asecodes sinense

Taxon classificationAnimaliaHymenopteraEulophidae

(Ling)

91C5125C-4C33-5262-9AC3-A6D0522EA18E

Fig. 17

Desmatocharis sinensis Ling, 2000: 260.

##### Material examined.

2♀ [NEFU; 1 on card, 1 on slide], China, Sichuan Province, Guangyuan City, Qingchuan County, 22.VIII.2015, Ye Chen and Chao Zhang, sweeping; 2♀ [NEFU; 1 on card, 1 on slide], CHINA, Yunnan Province, Lvchun County, Huanglianshan Natural Reserve, 18.I.2019, Jun-Jie Fan, Jun Wu and Ting-Ting Zhao, sweeping.

##### Diagnosis.

**Female.** Scape compressed, pedicel slightly shorter than half the length of F1; mesoscutellum with anterior 2/3 reticulated, posterior 1/3 smooth and shiny; fore wing with an infuscate transverse band below MV; metasoma longer than head plus mesosoma (ratio length of: metasoma : head : mesosoma about 5.2:1:3.5); gaster oblong ovate, about 2.2 times as long as its maximum width.

##### Host.

Unknown.

##### Distribution.

China (Yunnan (new record) and Sichuan (Ling 2000) Provinces).

##### Comments.

The original description of *Asecodes sinense* was given by Ling (2000). This species is similar to *A. turcicum* in having the fore wing with an infuscate transverse band below MV; mesoscutellum with anterior 2/3 reticulated, posterior 1/3 smooth and shiny. It can be separated from *A. turcicum* by its oblong ovate gaster, which distinctly longer than mesosoma (metasoma subequal to mesosoma in *A. turcicum*).

**Figure 17.**
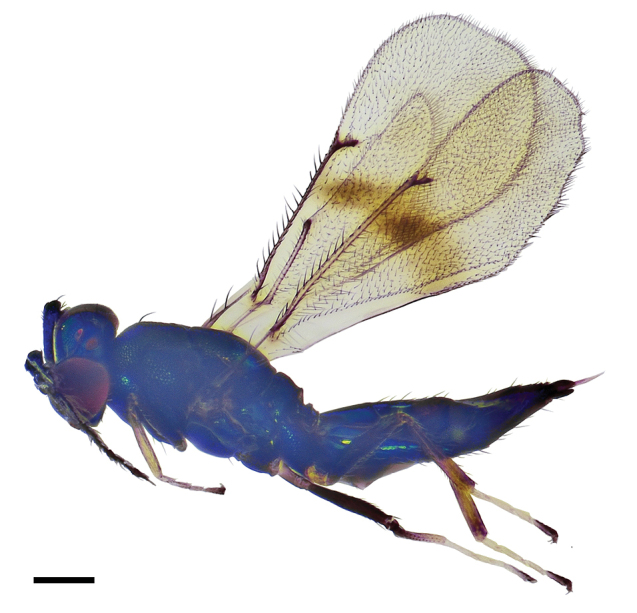
*Asecodes sinense* (Ling), female **17** habitus in lateral view. Scale bar: 200 μm.

#### Asecodes turcicum

Taxon classificationAnimaliaHymenopteraEulophidae

(Nees)

61CC48A8-81BF-5162-9E5C-424FFD5BB33D

Fig. 18

Asecodes turcicus (Nees): Hansson 1996: 162.
Closterocerus turcicus (Nees): Gumovsky 2003: 32.
Desmatocharis turcica (Nees): Graham 1959: 199.
Desmatocharis turcicus (Nees): Schauff 1991: 47.
Entedon turcicus (Nees): Walker 1839: 23.
Eulophus turcicus Nees, 1834: 155.
Teleopterus turcicus (Nees): Hansson 1994: 669.

##### Material examined.

2♀ [NEFU; 1 on card, 1 on slide], China, Tibet, Medog County (altitude: 1400 m), 22–29.VI. 017, Zhaxi, by Malaise trap; 3♀ [NEFU; 2 on cards, 1 on slide], CHINA, Tibet, Medog County (altitude: 1400 m), 6–13.VII.2017, Zhaxi, by Malaise trap.

##### Diagnosis.

**Female.** Scape compressed; mesoscutellum with anterior 2/3 reticulated, posterior 1/3 smooth and shiny; fore wing hyaline with an infuscate transverse band below MV; metasoma subequal to mesosoma, shorter than head plus mesosoma (ratio length of: metasoma : head : mesosoma about 3.2 : 1 : 3); gaster ovate.

##### Host.

Unkonwn.

##### Distribution.

China (Tibet (new record), Gansu (Zhang et al. 2007) and Sichuan (Ling 2000) Provinces), Japan (Kamijo 1986), Russia (Gumovsky 2003), India (Gumovsky 2003), Germany (Nees 1834), Czechoslovakia, France, Ireland (north and south), United Kingdom, Moldova (Bouček and Askew 1968), Netherlands (Gijswijt 2003), Sweden (Hansson 1991), Czech Republic (Kalina 1989).

##### Comments.

See Nees (1834) for the original description, and Jamali et al. (2021) for the photographs of the neotype of *Asecodes turcicum*.

**Figure 18.**
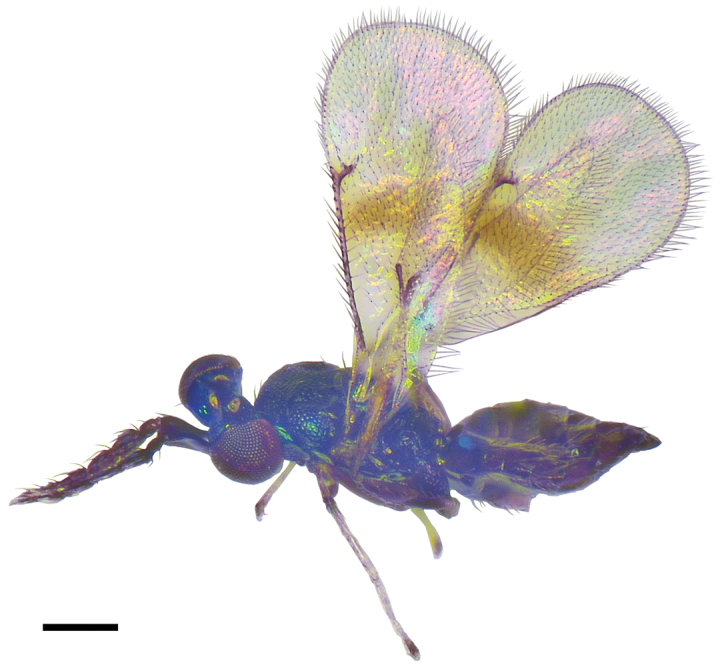
*Asecodes turcicum* (Nees), female **18** habitus in lateral view. Scale bar: 200 μm.

#### Asecodes delucchii

Taxon classificationAnimaliaHymenopteraEulophidae

(Bouček)

A8C38CDF-FB33-583C-A077-C2906E48DD73

Figs 19 , 20

Asecodes delucchii (Bouček): Hansson 1996: 162.
Asecodes deluchii (Bouček): Supartha and Ridland 2004: 3668 (misspelling).
Chrysocharoidea sp.: Graham 1963: 269.
Omphale sp.: Delucchi 1958: 241.
Teleopterus delucchii Bouček, 1971: 537.

##### Material examined.

4♀ [NEFU; 2 on cards, 2 on slides], China, Guizhou Province, Zunyi City, Suiyang County, 6.VIII.2020, Jun Wu, sweeping.

##### Diagnosis.

**Female.** Scape normal, not compressed; fore wing hyaline, without infuscate transverse band, and with three stigmal hairlines: two stigmal hairlines toward the apex of wing and one towards parastigma (Fig. 20).

**Figures 19, 20.**
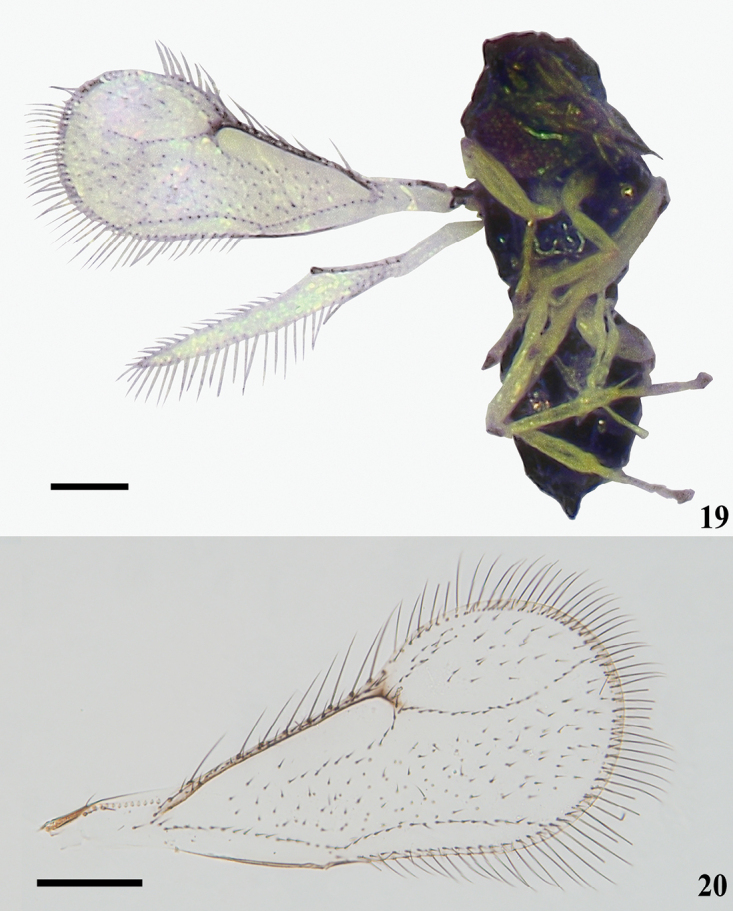
*Asecodes delucchii* (Bouček), females **19** habitus in ventral view **20** fore wing. Scale bars: 100 μm.

##### Host.

Primary parasitoid of the peach leafminer, *Lyonetia clerckella* (Linnaeus) (Lepidoptera, Lyonetiidae) (Adachi 1998) and the citrus leafminer *Phyllocnistis citrella* Stainton (Lepidoptera, Phyllocnistidae) (Ujiye and Adachi 1995).

##### Distribution.

China (Guizhou (new record) and Gansu (Zhang et al. 2007) Provinces), Japan (Adachi 1998), India (Jamali et al. 2021), Indonesia (Supartha and Ridland 2004), Croatia (Bouček 1977), Czechoslovakia, Italy, Poland, United Kingdom, Yugoslavia (pre-1991), Moldova (Bouček 1971), Romania (Hansson 2016).

##### Comments.

*Asecodes delucchii* can be easily separated from other species distributed in China by its characteristic fore wing. An Indian species, *A. zhui* Jamali having a similar fore wing was described by Jamali et al. (2021). *Asecodes delucchii* differs from *A. zhui* in having the fore wing about 2.4 times as long as wide (fore wing more than three times as long as wide in *A. zhui*); with the longest marginal cilia 1/3–1/2 the maximum wing width (4/5 the maximum wing width in *A. zhui*).

**Figures 21, 22.**
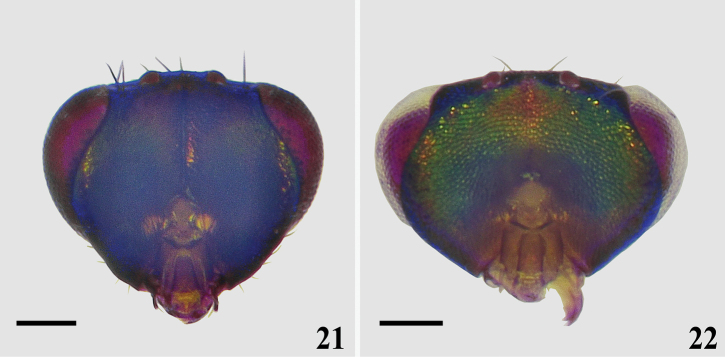
Head, showing occiput, females **21***Asecodes sinense* (Ling) **22***Closterocerus* sp. Scale bars: 100 μm.

## Supplementary Material

XML Treatment for Asecodes medogense

XML Treatment for Asecodes reticulatum

XML Treatment for Asecodes sinense

XML Treatment for Asecodes turcicum

XML Treatment for Asecodes delucchii
